# Malignant Clear Cell Hidradenoma of the Breast

**DOI:** 10.7759/cureus.1064

**Published:** 2017-03-01

**Authors:** Isaac Chambers, Ahmad K. Rahal, Pavan S Reddy, K. James Kallail

**Affiliations:** 1 Internal Medicine, University of Kansas School of Medicine-Wichita; 2 Hematology and Oncology, Baylor College of Medicine

**Keywords:** hidradenocarcinoma, myoepithelioma, acrospiroma, clear cell, hidradenoma, malignant, eccrine, apocrine, adenomyoepithelioma

## Abstract

A 58-year-old female had a mass in the right breast palpable beneath the areola. A mammogram revealed a 1.5-centimeter soft tissue density that was confirmed with a subsequent ultrasound. The patient underwent a core needle biopsy which was initially reported as a moderately differentiated invasive ductal carcinoma. Immunohistochemical analysis revealed negative staining for estrogen receptor (ER), progesterone receptor (PR), human epidermal growth factor receptor (HER2), mammaglobin, and gross cystic disease fluid protein 15 (GCDFP-15). A wide local excision of the mass was performed. The pathology report stated the tumor had an infiltrative growth pattern with a desmoplastic stromal response with enhanced epithelial atypia consistent with malignant transformation of a nodular clear cell hidradenoma. Clear cell hidradenoma is a very rare tumor originating from the sweat gland and has a propensity for the face and extremities. The malignant variant of this tumor is extremely rare and has been reported to originate from the breast in few cases. This case represents the difficulty in diagnosing this tumor along with the radiographic and histologic features that can distinguish this malignancy from other entities.

## Introduction

Clear cell hidradenomas are uncommon and very slow-growing tumors that originate from the sweat gland. These neoplasms have been considered to originate from the eccrine gland, but can also have apocrine differentiation. Only rarely have these tumors been identified in the breast, with only 18 reported cases in the literature [1]. Due to the rarity of the tumor, it is often a diagnostic challenge and only confirmed after complete excision. In extremely rare cases, hidradenomas are found to be malignant and are described in the literature using different nomenclature including clear cell hidradenocarcinoma, malignant clear cell myoepithelioma, malignant clear cell hidradenoma, clear cell eccrine carcinoma, and malignant clear cell acrospiroma as a few examples [2]. There are less than 50 reported cases in the literature of this malignant variant which are typically found on the face, scalp, or anterior surface of the trunk [3]. Accounting for all clear cell carcinomas, that makeup just 1.4% to 3% of all breast tumors. Clear cell hidradenocarcinoma originating from the breast is exceedingly rare with only a few reported cases in the literature [4-5]. The written consent was given by the patient.

## Case presentation

A 58-year-old female, nonsmoker presented to her primary care physician for a well woman exam. Her past medical history was significant for malignant melanoma of the skin, 20 years prior that was excised surgically. She was on hormone replacement therapy for a total of two years after menopause. Her family history was significant for breast cancer in her mother diagnosed at age 51.

**Figure 1:**
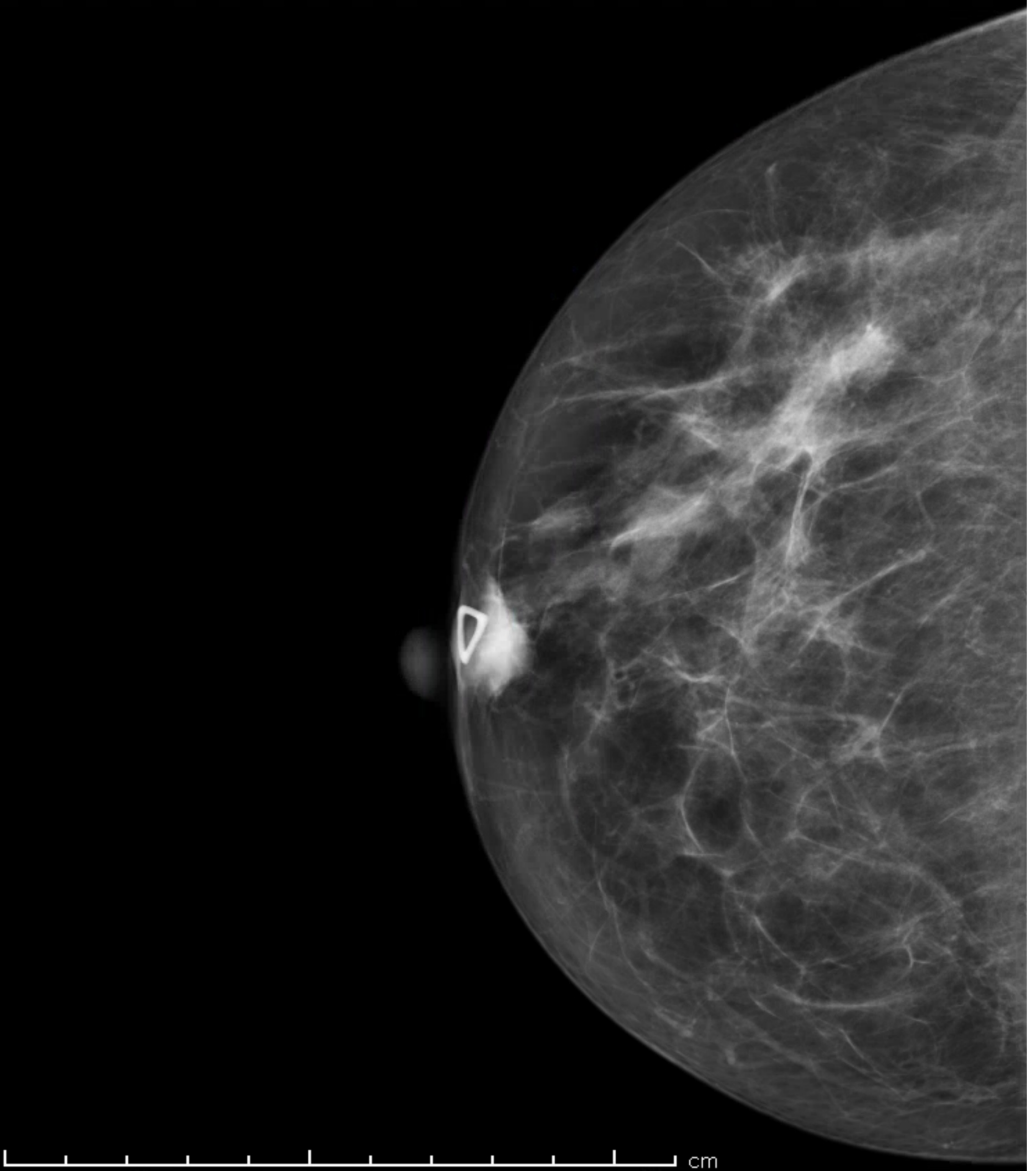
Diagnostic mammogram

On physical exam, a mobile and firm one-centimeter right breast mass were palpated without any associated lymphadenopathy. No breast tenderness or nipple discharge was reported. The patient underwent diagnostic mammography which revealed a breast imaging reporting and data system (BI-RADS) category four, 1.5-centimeter soft tissue density along the inferior portion of the areola of the right breast (Figure 1). A subsequent breast ultrasound showed a well-circumscribed 1.6-centimeter density with mixed signals, a considerable solid component, and some hypervascularity in the solid component.

A core needle biopsy of this lesion initially was reported to be a grade two invasive ductal carcinoma with focal metaplastic carcinoma and squamoid features. Unlike classical invasive ductal carcinoma, the neoplasm formed unusual broadsheets of neoplastic cells with pseudo-rosette formation and entrapped hyalinized vascular structures, some of which also showed myxoid degeneration. Immunohistochemical analysis revealed negative staining for estrogen receptor (ER), progesterone receptor (PR), and human epidermal growth factor receptor (HER-2) overexpression. Due to the proximity of the tumor to the skin, further immunostains including mammaglobin and gross cystic disease fluid protein 15 (GCDFP-15) were performed and were negative. The results favored a skin adnexal neoplasm most consistent with low-grade adnexal carcinoma of sweat gland origin.

**Figure 2:**
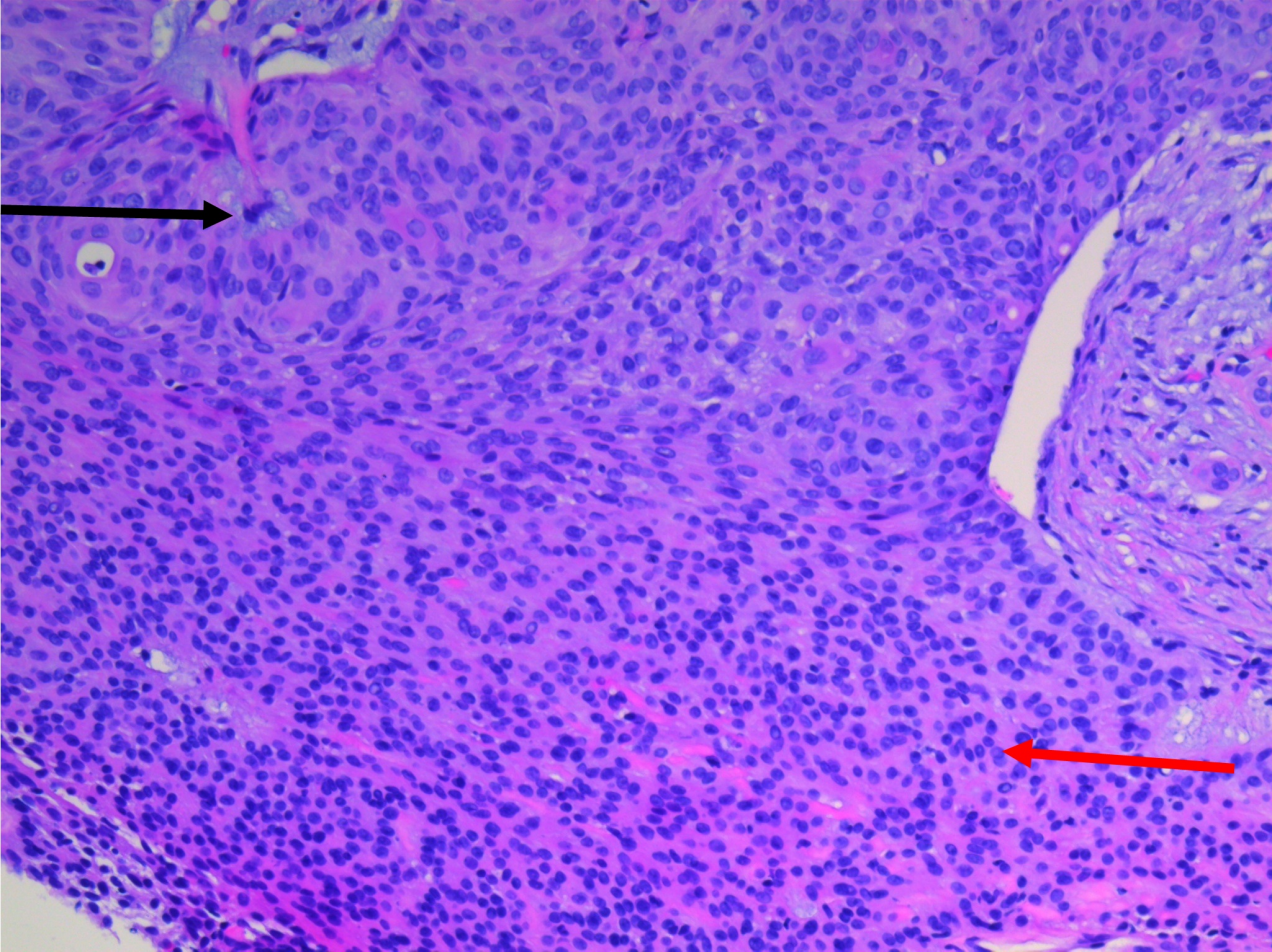
Biopsy of right breast, slide one The tumor is characterized by well differentiated cuboidal to squamoid appearing epithelial cells with focal areas of clear cell change (black arrow). There is a monotonous infiltrate throughout the neoplasm and significant duct formation (red arrow) including areas where the cells acquired more abundantly clear to the lightly eosinophilic cytoplasm

**Figure 3:**
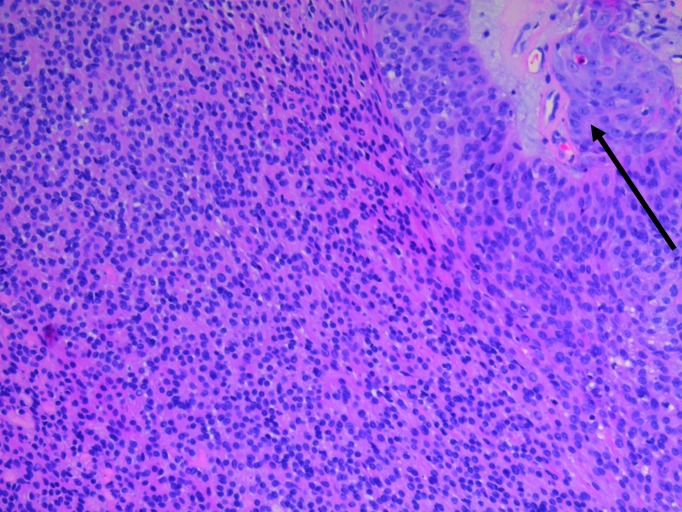
Biopsy of right breast, slide two The periphery of the neoplasm exhibited an infiltrative growth pattern with pronounced cytologic atypia (black arrow) and extension into the adjacent subcutaneous fat

**Figure 4:**
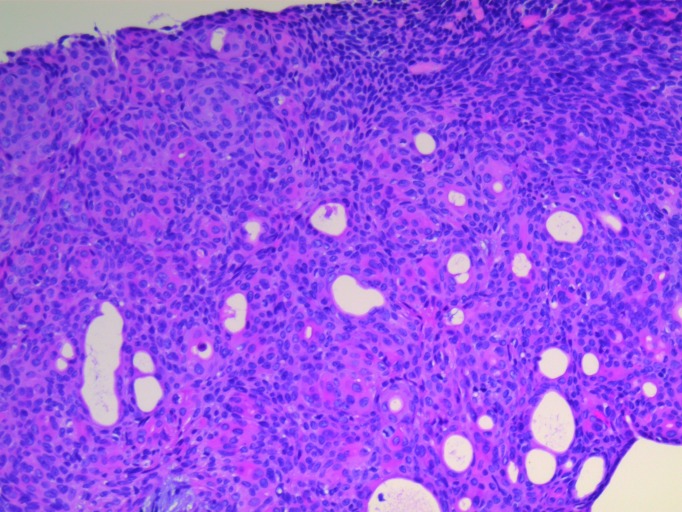
Biopsy of right breast, slide three Sheets of fused gland-like structures can be seen infiltrating through the fat

The patient underwent a lumpectomy with a 5.5 x 3.0 x 2.8-centimeters specimen removed, which contained an ellipse of skin measuring 5.0 x 2.5 centimeters. On gross examination, a partially cystic, tan-colored mass measuring 1.5 x 1.0 centimeters was found just beneath the skin surface with negative margins. Most of the tumor was well-differentiated (Figure 1); however, the periphery of the neoplasm exhibited an infiltrative growth pattern with enhanced cytologic atypia and extension into the adjacent subcutaneous fat. The proliferation index was around 10%-15%, with the Ki-67 staining being more prominent in the areas of infiltrative growth. In addition, there was extensive staining for tumor protein (p63). The histologic examination was consistent with a nodular clear cell hidradenoma originating from the eccrine apparatus that had undergone malignant transformation (Figures 2-4).

## Discussion

Clear cell hidradenoma of the breast is a rare tumor with only 18 reported cases in the literature, with the malignant variant being extremely rare [1,3]. The benign variant of the tumor commonly occurs in middle-aged women but the malignant form has no age or gender tendency [3]. Although most of these tumors are in the nipple or subareolar tissues, they can also arise from deep breast tissue. Because the incidence is low, these tumors are rarely reported based on radiological findings. Similar to our case, clear cell hidradenomas tend to be well-circumscribed and cystic with a variable solid portion and hypervascularity on ultrasound imaging [6].

Histologically, clear cell hidradenomas have a typical two-cell pattern of proliferation [7]. The first subpopulation of cells consists of polygonal cells with round nuclei with clear cytoplasm as the result of abundant glycogen. The second subpopulation of cells consists of cells with oval nuclei and dark basophilic cytoplasm that line the periphery of the duct structure [3,7]. Infiltrative growth patterns, frequent mitosis as well as perineural and angiolymphatic invasion are features that characterize the malignant variant of hidradenoma [2-3]. In contrast to adenocarcinoma of the breast, markers such as mammaglobin, GCDFP-15, ER, and PR will be negative and hidradenomas will usually stain positive for p63. The HER-2 staining has also proved to be useful, as less than 0.5% of cutaneous eccrine and apocrine neoplasms will stain positive, compared to 20%-35% of breast adenocarcinomas. Myoepithelial antibodies such as calponin, CD10, and S100 would be negative and assist in differentiating from an adenomyoepithelioma [1].

Malignant forms of clear cell hidradenoma typically arise de novo, but on occasion transform from the benign variant [5]. No features to date have been predictive of aggressive behavior [8]. Malignant variants tend to have an aggressive course with local recurrence around 50% and metastases in 60% of cases. Metastases tend to affect the regional lymph nodes, but can also involve the lung, bone, brain, liver, viscera, and skin [5,8-9]. Distant metastases are indicative of poor prognosis [8-9]. There are several other tumors with clear cell characteristics which can occur in the breast, including renal cell carcinoma, making it crucial to distinguish between a primary breast lesion and a breast metastasis [4].

Complete surgical excision with safe margins remains the treatment of choice for malignant clear cell hidradenoma [10]. Sentinel lymph node biopsy and elective regional lymphadenectomy may play a role in treatment, but this remains controversial [3,9]. There are reports of using trastuzumab for HER-2 positive tumors as well as hormonal therapy with some anecdotal success. Adjuvant chemotherapy and radiotherapy are not beneficial in local control or survival [3,5-9].

## Conclusions

Malignant clear cell hidradenoma is a rare oncologic entity and has only rarely been reported to occur in the breast. The rarity of the tumor, inconsistent nomenclature and histologic similarities with other tumors creates a diagnostic challenge [2,4]. It is important to consider clear cell hidradenomas and other sweat gland tumors when there are representative radiologic and histologic features. This case demonstrated that further immunostaining using breast markers is beneficial if there is diagnostic uncertainty.

## References

[1] Orsaria M, Mariuzzi L (2013). Recurrent eccrine hidradenoma of the breast in a male patient: problems in differential diagnosis. Our Dermatol Online.

[2] Pahar-Marinsek Z, Lamovec J (2003). Clear cell hidradenocarcinoma. Acta Dermatoven.

[3] Liapakis IE, Korkolis DP, Koutsoumbi A, Fida A, Kokkalis G, Vassilopoulos PP (2006). Malignant hidradenoma: a report of two cases and review of the literature. Anticancer Res.

[4] Ratti V, Pagani O (2015). Clear cell carcinoma of the breast: a rare breast cancer subtype - case report and literature review. Case Rep Oncol.

[5] Mezzabotta M, Declich P, Cardarelli M (2012). Clear cell hidradenocarcinoma of the breast: a very rare breast skin tumor. Tumori.

[6] Cho KE, Son EJ, Kim JA (2010). Clear cell hidradenoma of the axilla: a case report with literature review. Korean J Radiol.

[7] Ohi Y, Umekita Y, Rai Y (2007). Clear cell hidradenoma of the breast: a case report with review of the literature. Breast Cancer.

[8] Knoedler D, Susnik B, Gonyo MB, Osipov V (2007). Giant apocrine hidradenoma of the breast. Breast J.

[9] Nash JW, Barrett TL, Kies M (2007). Metastatic hidradenocarcinoma with demonstration of Her-2/neu gene amplification by fluorescence in situ hybridization: potential treatment implications. J Cutan Pathol.

[10] Sehgal S, Goyal P, Ghosh S (2014). Clear cell hidradenoma of breast mimicking atypical breast lesion: a diagnostic pitfall in breast cytology. Rare Tumors.

